# Assessment of Training Outcomes of Nurse Readers for Diabetic Retinopathy Telescreening: Validation Study

**DOI:** 10.2196/17309

**Published:** 2020-04-07

**Authors:** Marie Carole Boucher, Michael Trong Duc Nguyen, Jenny Qian

**Affiliations:** 1 Maisonneuve-Rosemont Ophthalmology University Center Department of Ophthalmology Université de Montréal Montreal, QC Canada; 2 Department of Ophthalmology Université de Montréal Montreal, QC Canada; 3 Department of Ophthalmology & Vision Sciences University of Toronto Toronto, ON Canada; 4 Hamilton Regional Eye Institute St Joseph's Healthcare Hamilton Hamilton, ON Canada; 5 Division of Ophthalmology Department of Surgery McMaster University Hamilton, ON Canada

**Keywords:** training, teleophthalmology, telemedicine, diabetic retinopathy, screening, referral, nurse

## Abstract

**Background:**

With the high prevalence of diabetic retinopathy and its significant visual consequences if untreated, timely identification and management of diabetic retinopathy is essential. Teleophthalmology programs have assisted in screening a large number of individuals at risk for vision loss from diabetic retinopathy. Training nonophthalmological readers to assess remote fundus images for diabetic retinopathy may further improve the efficiency of such programs.

**Objective:**

This study aimed to evaluate the performance, safety implications, and progress of 2 ophthalmology nurses trained to read and assess diabetic retinopathy fundus images within a hospital diabetic retinopathy telescreening program.

**Methods:**

In this retrospective interobserver study, 2 ophthalmology nurses followed a specific training program within a hospital diabetic retinopathy telescreening program and were trained to assess diabetic retinopathy images at 2 levels of intervention: detection of diabetic retinopathy (level 1) and identification of referable disease (level 2). The reliability of the assessment by level 1−trained readers in 266 patients and of the identification of patients at risk of vision loss from diabetic retinopathy by level 2−trained readers in 559 more patients were measured. The learning curve, sensitivity, and specificity of the readings were evaluated using a group consensus gold standard.

**Results:**

An almost perfect agreement was measured in identifying the presence of diabetic retinopathy in both level 1 readers (κ=0.86 and 0.80) and in identifying referable diabetic retinopathy by level 2 readers (κ=0.80 and 0.83). At least substantial agreement was measured in the level 2 readers for macular edema (κ=0.79 and 0.88) for all eyes. Good screening threshold sensitivities and specificities were obtained for all level readers, with sensitivities of 90.6% and 96.9% and specificities of 95.1% and 85.1% for level 1 readers (readers A and B) and with sensitivities of 86.8% and 91.2% and specificities of 91.7% and 97.0% for level 2 readers (readers A and B). This performance was achieved immediately after training and remained stable throughout the study.

**Conclusions:**

Notwithstanding the small number of trained readers, this study validates the screening performance of level 1 and level 2 diabetic retinopathy readers within this training program, emphasizing practical experience, and allows the establishment of an ongoing assessment clinic. This highlights the importance of supervised, hands-on experience and may help set parameters to further calibrate the training of diabetic retinopathy readers for safe screening programs.

## Introduction

### Diabetic Retinopathy and Remote Screening

Diabetic retinopathy is the main cause of legal and functional blindness in the working-age population and in many developed countries [1,2]. Timely identification of individuals with diabetes who are at risk [3] and early management of diabetic retinopathy significantly reduces the progression to blindness [4].

The use of teleophthalmology programs to detect diabetic retinopathy and manage follow-up has been shown to be cost-effective [5] and valuable [6-9]. However, there are also concerns about accurate diagnosis and treatment decisions by retina specialists or ophthalmologists [7,10-20]. Family physicians trained to assess diabetic retinopathy have shown good levels of agreement with retina specialists [21-23]. In an attempt to improve resource management and relieve the reading interpretation burden on ophthalmologists, various diabetic retinopathy screening programs have introduced nonphysician trained graders to identify patients at risk of vision loss from diabetic retinopathy [23-29]. Previous studies have discussed the sensitivity of human graders for referable disease [30,31] and the workload required for graders to maintain expertise [32]. However, the literature is scant on specific reader training, involving only small numbers of trainees [33], and outcomes are evaluated without training specifications [34]. To our knowledge, other than the UK training program [35], there is no set minimum practical experience required for training diabetic retinopathy readers, and none that specifically addresses the performance curve with training experience.

### Study Objectives

This study aimed to evaluate the performance, safety implications, and progress of 2 ophthalmology nurses in detecting diabetic retinopathy and identifying referable diseases following specific training in a diabetic retinopathy telescreening program. Their reading results were compared with those obtained from a retina specialist and the gold standard, consisting of a group-arbitrated consensus. A secondary objective was to determine the reason for reading discrepancies.

This study identifies training parameters to help tailor and standardize the training of nonophthalmologist readers for safe diabetic retinopathy interpretation in a screening program and validates the individual and group performance of trainee readers within this program. However, as with any screening program, the need for continuous monitoring and education of readers after the training process remains necessary.

## Methods

### Ethical Considerations

This study is approved by the Institutional Suitability Committee, the Scientific Evaluation Committee and the Research Ethics Committee of the Centre Intégré Universitaire de Santé et de Services Sociaux de l'Est-de-l'Île-de-Montréal, Montreal, Québec, Canada, where it was conducted (US Federal Wide Assurance numbers FWA00001935 and IRB00002087).

### Study Population, Design, and Data Collection

This retrospective interobserver reliability study was conducted on 829 patients with type 2 diabetes who attended a screening visit within a hospital-based teleophthalmology program at the Maisonneuve-Rosemont University Ophthalmology Center between February 2016 and September 2018. A total of 4 patients with laser scars from diabetic retinopathy treatment were mistakenly included in the program, who were excluded from the analysis; therefore, the final analysis was conducted on 825 individuals (1650 eyes). Patients were imaged by an ophthalmic photographer with a nonmydriatic camera (iCam-Optovue) after pupil dilation with 1% tropicamide to reduce ungradable imaging. Two 45-degree image fields, 1 image centered on the disc and 2 centered on the macula, were obtained to ensure adequate macular imaging. Demographics were not collected.

The images were securely transmitted to a dedicated hospital server and accessed by all readers from a teleophthalmology diabetic retinopathy electronic platform (iVision from RetinaLabs), which allowed interpretation by various levels of readers. The images were reviewed nonstereoscopically at the capture resolution, with automated or manual image enhancement (magnification, brightness, and contrast) (Adobe Photoshop 7.0, Adobe Systems Inc). Images were assessed using a grading software that showed the grading scheme and the Early Treatment Diabetic Retinopathy Study (ETDRS) standard photographs as references at all times. The integrated grading scheme is based on the Scottish Diabetic Retinopathy Grading Scheme (2007) [36] described in Multimedia Appendix 1, which resembles that of the American Academy of Ophthalmology. It takes into account two 45-degree imaging fields and refers to the ETDRS standard photographs. In this program, the absence of any diabetic retinopathy leads to a 2-year imaging recommendation.

Through the teleophthalmology platform, level 1 readers determine for each eye, the image quality, if diabetic retinopathy is present (corresponding to ≥R1) or absent, and identify any other detected abnormalities. Level 2 readers determine image quality and grade diabetic retinopathy in 5 severity levels: no retinopathy (R0), mild (R1), moderate (R2), severe nonproliferative diabetic retinopathy (R3), and proliferative diabetic retinopathy (R4). They also specifically grade diabetic macular edema (DME) as none (M0), presence of any microaneurysm, hemorrhage, or exudate within 2 disc diameters (DD) of the fovea (M1), or within 1 DD of the fovea (M2). Any other abnormality was identified for ophthalmologic attention as well. Ungradable images are labeled as R6 for the general diabetic assessment and M6 for the macular assessment by all readers, which leads to an automatic referral for an in-person examination after validation by the level 3 reader (retina specialist). The level 3 reader (MB), who is blinded to the trained readers, rereads all of the images on the same teleophthalmology platform, acting as a level 1 or level 2 reader.

For teaching and quality assurance purposes, a weekly group review was attended by all 3 readers, where any discrepancies of level 1 or 2 readings with that of the level 3 reader generated by the built-in quality assurance module of the electronic reading platform, were discussed. The final consensus of any reading disagreements was determined by group arbitration, which was established as the gold standard.

### Training of the Readers

Two ophthalmology nurses (A and B), 1 technical and 1 clinical, voluntarily participated in this study and were trained successively to intervene as level 1 and level 2 readers. Outside of training for visual acuity measurement and instillation of dilating eye drops, they had no relevant experience or credentials in assessing diabetic retinopathy or prior involvement in any eye imaging.

The training of level 1 reading was provided by a validated interactive electronic platform [37] assuming no prior knowledge or background on diabetic retinopathy. The platform teaches the characteristic features of normal fundi, those of diabetic retinopathy, and the recognition of image quality. It allows the graders to grade in one or multiple sessions and lasts a total of about 3 hours. The training is concluded by a self-assessment quiz on 50 diabetic patients (100 eyes), of which 60% (30/50) had some diabetic retinopathy and were further subdivided as 80% (24/30) R1, 10% (3/30) R2, 3% (1/30) R3, and 6% (2/30) R4; 28% (14/50) had no diabetic retinopathy and 12% (6/50) showed insufficient image quality to allow reading. The self-assessment is performed in 1 session without any time limit, although it generally lasts about 2 hours. An arbitrary 80% success threshold allows access to level 1 reading with ongoing quality control by the retina specialist.

Training for level 2 reading involves weekly sessions of quality assurance and group reviews of all new level 1 individual readings. This enables progressive recognition of diabetic retinopathy severity, which leads to a referral to a level 3 reader (retina specialist if the severity is > R2 (exceeds a moderate level of retinopathy) or ≥ M2 (possible DME within 1 DD from the fovea. The precautionary referral of any uncertain or unusual findings, such as other pathology or atypical variation of normal characteristics, is emphasized.

The level 1 readers spontaneously reported feeling comfortable for level 2 reading after the group review and training on 266 imaged patients (532 eyes), of which reader A had individually assessed 114 patients and reader B, 152 patients. This was set as the starting point for the evaluation of their next readings for a total of 1118 level 2 eye readings in 559 patients (323 patients for reader A and 236 for reader B).

### Statistical Analysis

The kappa (*κ*) statistic based on the Landis and Koch system [38] evaluates the reliability of the assessment beyond that of chance for the level 1 and level 2 readings in all readers against the consensus gold standard. It also evaluates the level 3 reader’s reliability for each level 1 and 2 cohort and to the gold standard; 95% CIs were used, and *P* values of <.001 were considered significant.

The screening performance (sensitivity and specificity), diagnostic accuracy (95% CI), and the learning curve in 50-patient strata of the level 1 and level 2 readers were calculated with the consensus gold standard readings as those of the level 3 reader with respect to each level 1 and level 2 cohorts. Grading of the most affected eye was used to calculate the sensitivity and specificity of the patient readings.

## Results

### Demographics

There were 532 eyes (266 patients) evaluated at the level 1 reading level, of which level 1 reader A and reader B individually assessed 228 eyes (114 patients) and 304 eyes (152 patients), respectively. A total of 1118 eyes (559 patients) were assessed by the level 2 readers, which also included an evaluation for DME, and of which level 2 reader A and reader B assessed 646 eyes (323 patients) and 472 eyes (236 patients), respectively.

Excluding any ungradable images as per the consensus gold standard, the global prevalence of diabetic retinopathy (≥R1) was 46.2% (117/254) and 37.3% (196/526) in the level 1 and level 2 cohorts, respectively, and the total prevalence of diabetic macular involvement was 25.8% (135/523). The prevalence and distribution of disease severity and number of ungradable images were comparable between level 1 and level 2 cohorts and between reader A and B according to the consensus gold standard grading (Multimedia Appendix 2). They were also comparable for diabetic retinopathy severity, DME, and ungradable imaging in each individual level reader (Multimedia Appendices 3-5).

### Referral Reasons

The most common reason for referral was DME (102/151, 67.5%), followed by severe diabetic retinopathy with DME (11/151, 7.3%), and severe diabetic retinopathy without DME (2/151, 1.3%; Table 1). DME represented 76% (70/92) and 57% (38/67) of the level 2 reader A and B referrals, respectively, and 72% (57/79) and 58% (36/62) of those of the retina specialist with respect to the level 2 reader images.

The kappa values in Table 2 show good agreement for referable disease in all eye readings and for all level readers. There is almost perfect agreement in identifying the presence of diabetic retinopathy by level 1 readers (κ=0.86 and 0.80) and in identifying referable disease (>R2) by level 2 readers (κ=0.80 and 0.83), compared with the gold standard. At least substantial agreement was measured in level 2 readers versus the gold standard for macular edema (M>1; κ=0.79 and 0.88) as well as for deciding if a referral to ophthalmology was warranted (*κ*=0.76 and 0.89). The level 3 reader, acting as a level 2 reader, achieved an almost perfect agreement with kappa values of 0.95, 0.95, and 0.95 for referable retinopathy, DME, and decision to refer to ophthalmology, respectively.

**Table 1:** Reasons for diabetic retinopathy referral in level 2 and level 3 readers and the consensus gold standard (N=559).

Diabetic retinopathy grading	Reader A, n (%)	Level 3 reader for reader A, n (%)	Consensus gold standard for reader A, n (%)	Reader B, n (%)	Level 3 reader for reader B, n (%)	Consensus gold standard for reader B, n (%)	Consensus gold standard for all readings, n (%)
M>1 only (including R6)	70 (76)	57 (72)	60 (72)	38 (57)	36 (58)	42 (62)	102 (67.6)
R6 and M6 only	16 (17)	17 (22)	17 (21)	18 (27)	19 (31)	19 (28)	36 (23.8)
R>2 and M>1	5 (5)	4 (5)	4 (5)	8 (12)	7 (11)	7 (10)	11 (7.3)
R>2 only (including M6)	1 (1)	1 (1)	2 (2)	4 (5)	0 (0)	0 (0)	2 (1.3)
Total referrals	92	79	83	67	62	68	151

**Table 2:** Agreements of level 1, 2, and 3 readings for referable (>R2) diabetic retinopathy and diabetic macular edema (>M1) and referral to ophthalmology for all eyes versus the consensus gold standard (level 1 reading [n=266] and level 2 reading [n=1118]).

Reader		Consensus gold standard referable diabetic retinopathy, κ^a^ (95% CI)	Consensus gold standard referable diabetic macular edema grading, κ (95% CI)	Consensus gold standard referral to ophthalmology, κ (95% CI)
**Level 1 reading (n=266)**				
	Reader A (n=114)	N/A^b^	N/A	0.859 (0.764-0.953)
	Level 3 reader for reader A	N/A	N/A	1.00 (1.000-1.000)
	Reader B (n=152)	N/A	N/A	0.803 (0.709-0.896)
	Level 3 reader for reader B	N/A	N/A	1.00 (1.000-1.000)
**Level 2 reading (n=1118)**				
	Reader A (n=646)	0.803 (0.757-0.850)	0.788 (0.733-0.842)	0.757 (0.677-0.838)
	Level 3 reader for reader A	0.940 (0.912-0.968)	0.961 (0.935-0.986)	0.967 (0.935-0.999)
	Reader B (n=472)	0.826 (0.777-0.874)	0.877 (0.830-0.925)	0.887 (0.822-0.952)
	Level 3 reader for reader B	0.957 (0.930-0.983)	0.946 (0.914-0.979)	0.936 (0.886-0.987)

^a^κ: kappa coefficient. All kappas have *P* values <.001.

^b^Not applicable.

### Reader Agreements and Referrals

With respect to the cohorts, good screening threshold sensitivities and specificities were obtained in all level readers (Table 3), with sensitivities of 91% and 97% and specificities of 95% and 85% for level 1 readers A and B, and sensitivities of 86.8% and 91.2% and specificities of 91.7% and 97.0% for level 2 readers. Reader B achieved slightly better sensitivities than reader A, and the level 3 reader achieved the highest sensitivity and specificity.

**Table 3:** Sensitivity and specificity for the identification of patient referrals by each reader versus the consensus gold standard.

Reader		Number of patients, n	Sensitivity, % (95% CI)	Specificity, % (95% CI)
**Level 1 reading (n=266)**				
	Reader A	114	91 (82.70-98.44)	95 (89.66-100.51)
	Level 3 reader for reader A	114	100 (100-100)	100 (100-100)
	Reader B	152	97 (92.72, 101.12)	85 (77.57-92.55)
	Level 3 reader for reader B	152	100 (100-100)	100 (100-100)
**Level 2 reading (n=559)**				
	Reader A	323	86.8 (79.45-94.04)	91.7 (88.17-95.16)
	Level 3 reader for reader A	323	95.2 (90.57-99.790)	100 (100-100)
	Reader B	236	91.2 (84.43-97.92)	97.0 (94.45-99.59)
	Level 3 reader for reader B	236	91.2 (84.43-97.92)	100 (100-100)

Level 2 and 3 reading discrepancies with the consensus gold standard and their impact on patient management are described in Table 4. Both level 2 readers show a higher overall patient disagreement rate with the consensus gold standard (66/323, 20.4% and 42/236, 17.8%) than the level 3 reader (18/323, 5.6% and 14/236, 5.9%), respectively, but a high proportion of the level 2 reader disagreements (57/66, 86% and 36/42, 86% respectively) had only minor or no impact on patient management.

A missed referral to ophthalmology is considered a significant misreading and occurred in 2.8% (9/323) and 2.5% (6/236) of patients in the level 2 readings, respectively, and respective to these cohorts, in 1.2% (4/323) and 2.5% (6/236) of patients in the level 3 readings. A comparable rate of significant misreading (excluding underappreciation of image quality) is shown in both level 2 readers (6/323, 1.9% and 5/236, 2.1%, respectively for reader A and reader B) and level 3 readers (4/323, 1.2% and 6/236, 2.5%). All image misreadings were related to unrecognized isolated microaneurysms located within 1 DD of the fovea in the absence of any exudate, except for 1 eye with neovascularization misinterpreted as an epiretinal membrane by the level 3 reader and confirmed on clinical examination.

Level 2 readers also show an overall underappreciation of ungradable imaging in 1.2% (4/323) and 0.8% (2/236) of the patients, respectively for reader A and reader B. Stratified analysis of 50 successive patients showed that as experience was gained, this rate was still maintained.

The consequences of misreading on patient management, such as the timing of new imaging or referral for in-person examination, were measured to be 73% (48/66) and 55% (23/42) in the level 2 reader cohorts, respectively, and in 67% (12/18) and 64% (9/14) of the level 3 reader, respectively, in the level 2 cohort.

Both level 2 readers tended to be more conservative in their actions, with 6.5% (21/323) and 2.1% (5/236) unnecessary referral recommendations, as compared with 0% for the level 3 reader, reimaging sooner than indicated in 4.3% (14/323) and 4.7% (11/236) of patients, respectively. Both level 2 readers acknowledged possible unnecessary referrals, but still referred patients as a precaution in 1.2% (4/323) and 0.4% (1/236) of all screenings, respectively, which represented 6% (4/66) and 2% (1/42) of their misreads.

**Table 4:** Level 2 and level 3 reader disagreements according to the consensus gold standard and impact on patient management (N=559).

Effect of disagreement		Reader A (n=323), n (%)		Level 3 reader for reader A (n=323), n (%)		Reader B (n=236), n (%)		Level 3 reader for reader B (n=236), n (%)	
No impact on patient management		18 (5.6)		6 (1.9)		19 (8.1)		5 (2.1)	
Impact on patient management		48 (14.9)		12 (3.7)		23 (9.8)		9 (3.8)	
Total number of disagreements		66 (20.4)		18 (5.6)		42 (17.8)		14 (5.9)	
No referral although indicated		9 (2.8)		4 (1.2)		6 (2.5)		6 (2.5)	
Unnecessary referral		21 (6.5)		0 (0)		5 (2.1)		0 (0)	
Imaging recommended sooner than necessary		14 (4.3)		0 (0)		11 (4.7)		1 (0.4)	
Imaging recommended later than indicated		4 (1.2)		8 (2.5)		1 (0.4)		2 (0.9)	
**Significant misreads (no referral although indicated)**									
	Missed isolated microaneurysm within 1 DD^a^ of the fovea.		6 (1.9)		3 (0.9)		5 (2.1)		6 (2.5)
	Confusion of neovascularization with an epiretinal membrane		0 (0)		1 (0.3)		0 (0)		0 (0)
	Under appreciation of ungradable imaging		3 (0.9)		0 (0)		1 (0.4)		0 (0)
**Nonsignificant misreads**									
	Misreads with minimal impact on management		34 (10.5)		8 (2.5)		15 (6.4)		3 (1.3)
	Referrals as a precaution		4 (1.2)		0 (0)		1 (0.4)		0 (0)
	Under appreciation of ungradable imaging		1 (0.3)		0 (0)		1 (0.4)		0 (0)

^a^DD: disc diameter.

### Learning Curve of Trained Readers

The per-strata sensitivities and specificities of level 1 and level 2 readers show high sensitivity and specificity for all readers, achieved immediately after training to detect any presence of diabetic retinopathy for level 1 readers and, for level 2 readers, to identify referable disease (>R2 and/or >M1), which were maintained throughout the study (Multimedia Appendices 6 and 7).

**Figure 1:**
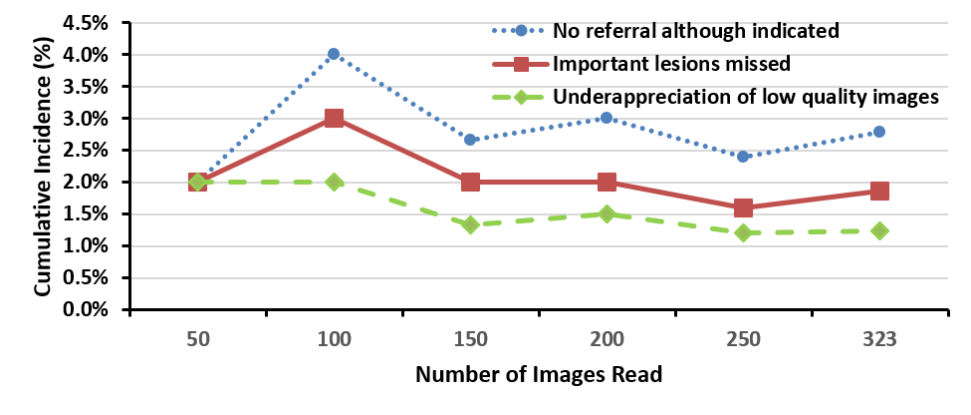
The cumulative incidence curve of misreadings for level 2 reader A image readings.

**Figure 2:**
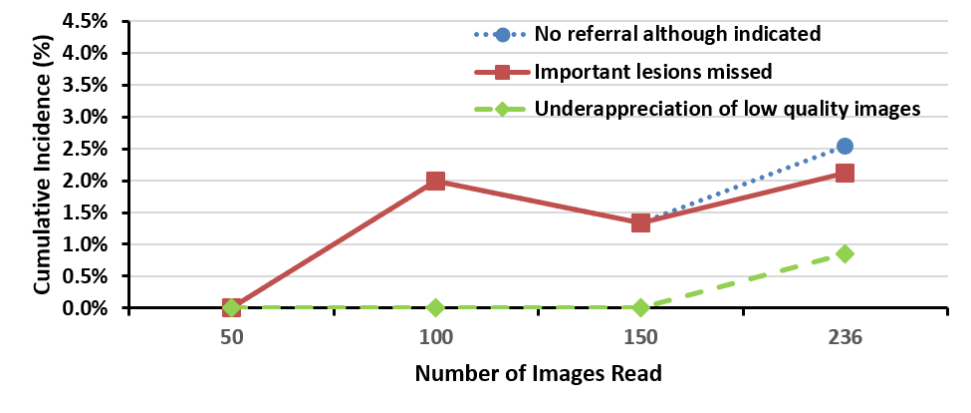
The cumulative incidence curve of misreadings for level 2 reader B image readings.

Figures 1 and 2 show the cumulative incidence of misreads with time and gained experience to be more related to specificity than sensitivity issues. The small number of disagreements in each stratum impedes the analysis of tendencies for the reasons for disagreements as more experience is gained.

## Discussion

### Principal Findings

This study emphasizes the importance of practical experience and validates the screening performance and training of level 1 and level 2 diabetic retinopathy readers within this program. It may thus help set parameters to further calibrate the training of diabetic retinopathy readers for safe screening programs.

It shows 91% and 97% sensitivities, and 95% and 85% specificities in detecting any diabetic retinopathy, and 86.8% and 91.2% sensitivities, and 91.7% and 97.0% specificities in the identification of sight-threatening disease relative to the cohorts. These results are comparable to those reported in studies with similar conditions [33,39-42]. There is substantial overall intergrader agreement obtained by the 2 level 2 readers across all grading episodes for all referable retinopathy (*κ*=0.757, 95% CI 0.677-0.838 and *κ*=0.887, 95% CI 0.822-0.952, respectively). Although inferior to those of the retina specialist (*κ=*0.967, 95% CI 0.935-0.999 and κ=0.936, 95% CI 0.886-0.987), they compare favorably with the results by Goatman et al (κ median 0.78, interquartile range 0.70-0.84) [42] who also used a consensus reading gold standard and similar diabetic retinopathy severity grading and outcome schemes and who achieved 95.3% sensitivities for referable diabetic retinopathy. In a quality assurance audit of 6 trained graders, Patra et al [43] found a strong agreement between graders and the retina specialist reference standard with a kappa of 0.7. This study’s kappa values were greater than those reported by Patra et al [43] and exceeded their 80% set audit standards for interobserver agreement.

Ruamviboonsuk et al [33] trained 3 reading photographers and 3 ophthalmology nurses in a 2-day course, which showed only fair agreement with the 3 retina specialists consensus group regarding retinopathy severity, macular edema, and referrals. They concluded that this course was insufficient to adequately train nonphysicians in the appropriate reading skills. In contrast, the practical training of this study is extensive, and the graders of the Bhargava et al study underwent a 1-year rigorous training with regular auditing [41]. It is noteworthy that the graders of our study showed a high appreciation of the quality assurance and teaching procedures in their training.

Although not consistently met in many studies evaluating gold standards in diabetic retinopathy detection [30], targets of 80% and 90% to 95% sensitivity and specificity are recommended for diabetic retinopathy assessment by trained examiners [44,45]. The challenge of finding an appropriate gold standard in the grading of diabetic retinopathy, especially in ambiguous gradings, was met in our study by establishing a group-consensus arbitration gold standard. Although differences in diabetic retinopathy grading systems and reference gold standards complicate the comparisons, the previous authors also found a strong agreement between the graders and the retina specialist reference standard and concluded that trained nonphysician graders can provide high levels of accuracy in diabetic retinopathy and maculopathy detection and assessment.

Certification training programs, such as that of the United Kingdom National Health Service, suggest that good reading performance indicates good training but does not address minimal practical training experience for readers [32,46]. This study addresses the latter and found that practical training of level 1 readers on a teaching electronic platform and self-assessment on 50 patients resulted in a high intergrader agreement and high sensitivity and specificity rates for detecting diabetic retinopathy and identifying ungradable images, approaching those of the retina specialist and gold standard. Further training for referable diabetic retinopathy and macular edema through a group review of 532 eyes in 266 patients led to an immediate high agreement and sensitivity and specificity for this task, which was maintained in the next readings of 646 eyes in 323 patients and 472 eyes in 236 patients, respectively. This may be used as a threshold for similar practical training experience for nonophthalmologist diabetic retinopathy graders to meet quality standards in similar individuals and settings.

The failure of level 2 readers to recognize inadequate imaging under pupil dilation in 1.2% (4/323) and 0.8% (2/236) of all readings, respectively, represented 6% (4/66) and 5% (2/42) of all of their disagreements with the gold standard. In comparison, Farley et al [22] showed that 5.2% of eyes with inadequate imaging failed to be referred by trained primary care clinician readers in a study with a high rate of inadequate imaging due to nondilating pupils (29%). Although the readers of this study were provided objective gradable image guidelines, possible borderline-quality images could have led to subjective assessments. Failure to recognize inadequate imaging underlines the importance of pursuing reader education and regular monitoring. The underappreciation of ungradable images in our study is in contrast with that of Ruamviboonsuk et al, who interpreted their high proportion of ungradable images as a lack of confidence in reading rather than true image ambiguity [33].

Level 2 readers made more conservative assessments, resulting in precautionary referrals in 1.2% (4/323) and 0.4% (1/236) of their readings versus none of the level 3 readings. Although these rates are small, further training to recognize unusual variants of normal and those having to be brought to the attention of the ophthalmologist as a precaution may help increase specificity and further reduce the workload on ophthalmologists.

Significant misreads causing missed referrals to ophthalmology were all related to missed isolated microaneurysms located within 1 DD of the fovea in the absence of any exudate, except for 1 level 3 reader misinterpretation of neovascularization as an epiretinal membrane. An isolated microaneurysm within 1 DD of the fovea does not signal DME unless associated with a positive optical coherence tomography establishing edema, but does signal a potential risk of DME with time. Missed detection of possible DME was found to be the worst scenario in 1.9% (6/323) and 2.1% (5/236) of level 2 reader significant misreads and in 0.9% (3/323) and 2.5% (6/236) of those of the level 3 reader. Level 2 readers appear to have greater sensitivity in detecting these isolated microaneurysms, as these misreadings represent 9% (6/66) and 12% (5/42) of all of their disagreements with the gold standard in comparison to 17% (3/18) and 36% (5/14) of those of level 3 respective to the cohorts. Moss et al [47] similarly showed that most disagreements with all level readers are related to the nondetection of isolated microaneurysms in very mild disease states.

DME was the major cause of referral in this study at 65% of all referrals, followed by 8.2% for severe diabetic retinopathy with DME and 1% for severe diabetic retinopathy without DME.

**Figure 3:**
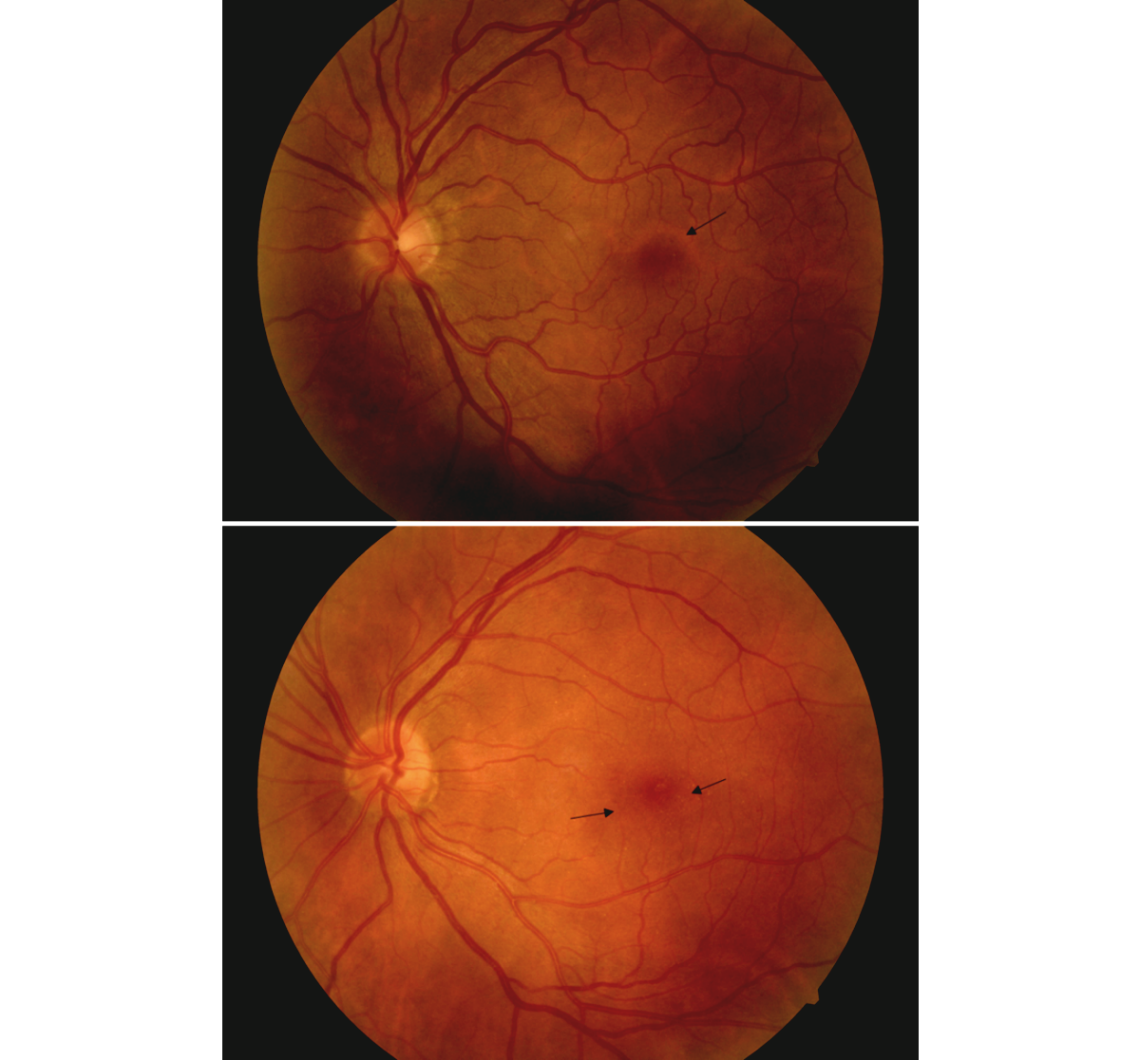
Two challenging cases of an isolated microaneurysm near the fovea. Arrows are used to indicate the location of microaneurysms.

Although overall screening posed no visual safety threat in 98.0% (548/559) of patients assessed by the level 2 readers (317/323, 98.1% and 231/236, 97.9%, respectively) and 98.2% (549/559) of all level 3 readings, a small number could be put at risk with this process. The majority were related to difficult positive identification of isolated microaneurysms in the macular area at the limit of detection, which often resulted in arbitration for the final gold standard grading. These could potentially and eventually be resolved with the use of greater resolution cameras for screening. Recommendations for reimaging later than required could represent some level of risk in 1.2% (4/323) and 0.4% (1/236) of the patients assessed by the level 2 readers compared with those of the level 3 reader. Figure 3 shows images of 2 challenging cases of an isolated microaneurysm near the fovea.

This study outperforms the screening results of Oke et al [48] showing that human readers miss 11% of sight-threatening diabetic retinopathy. They also conclude that low-grade-diabetic retinopathy misclassification is not uncommon but unlikely to lead to significant referral delays in sight-threatening diabetic retinopathy. The management of the small number of patients in whom a significant lesion is missed in 1 eye is also dependent upon the presence of other abnormalities in that eye or the other eye. As such, it cannot be shown if these patients would be referred had these lesions been present in an isolated state.

### Limitations

Limitations of this study include its retrospective nature and the small number of trained readers, which only validates the individual and group performance of these readers within this specific training. These results may not apply to a larger reading group where possible individual performance variations could occur.

### Conclusions

This study validates the screening performance and accuracy of the specific training of 2 nonphysician graders as level 1 (triage) and level 2 (referable diabetic retinopathy) graders who achieved a very high initial agreement that was maintained throughout the study and whose image interpretations compared favorably with that of a retina specialist and the consensus gold standard. It adds new information to scant literature on diabetic retinopathy reader training modalities, emphasizes the importance of training experience for reading, and suggests a starting threshold in a similar setting to train nonophthalmologist readers and meet quality standards. As with other studies [39,49], it supports the need for continual performance monitoring and education of diabetic retinopathy readers after their training to guarantee ongoing high standards expected in any diabetic retinopathy screening service. Although this study allows the establishment of an ongoing diabetic retinopathy assessment clinic with these readers, it only describes the results of 2 individual readers and possible significant individual performance variations could occur in larger trainee groups.

## Appendix

Multimedia Appendix 1 Summary of the Scottish Diabetic Retinopathy Grading Scheme 2007 v1.1.

Multimedia Appendix 2 Patient distribution of disease severity according to the worst eye and ungradable imaging between reader A and B, according to gold standard grading.

Multimedia Appendix 3 Diabetic retinopathy findings and ungradable imaging in level 1 and level 3 readers and in the consensus gold standard.

Multimedia Appendix 4 Findings and prevalence of diabetic retinopathy severity, diabetic macular edema, and ungradable imaging in patients by individual readers in level 2 and level 3 readings and the consensus gold standard.

Multimedia Appendix 5 Findings and prevalence of diabetic retinopathy severity, diabetic macular edema, and ungradable imaging by individual readers for all eyes imaged in level 2 and level 3 readings and the consensus gold standard.

Multimedia Appendix 6 Per-strata sensitivities and specificities of level 2 readers for every patient as determined by the eye with the worst grading severity.

Multimedia Appendix 7 Per-strata sensitivities and specificities of level 2 readers for all eyes.

## Abbreviations

DD: disc diameter
DME: diabetic macular edema
ETDRS: Early Treatment Diabetic Retinopathy Study
