# Supplementary figures and images for: Assessment of Training Outcomes of Nurse Readers for Diabetic Retinopathy Telescreening: Validation Study
Source: JMIR Diabetes. 2020 Apr 7;5(2):e17309. doi: 10.2196/17309 (PMC7175194; doi:10.2196/17309)

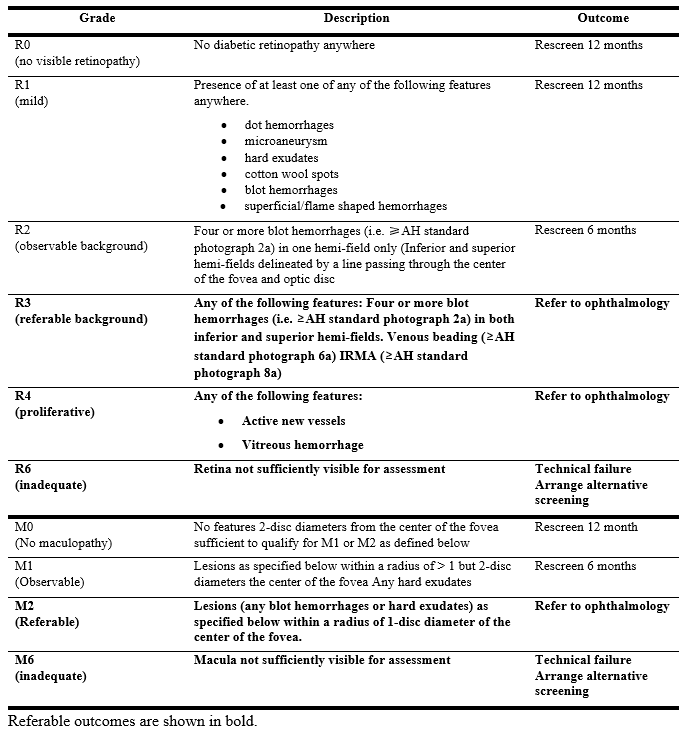

Supplement: Multimedia Appendix 1 [file diabetes_v5i2e17309_app1.PNG]

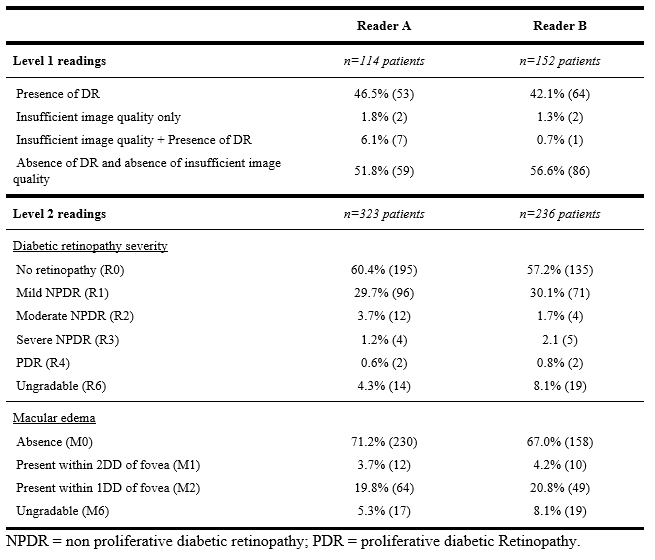

Supplement: Multimedia Appendix 2 [file diabetes_v5i2e17309_app2.PNG]

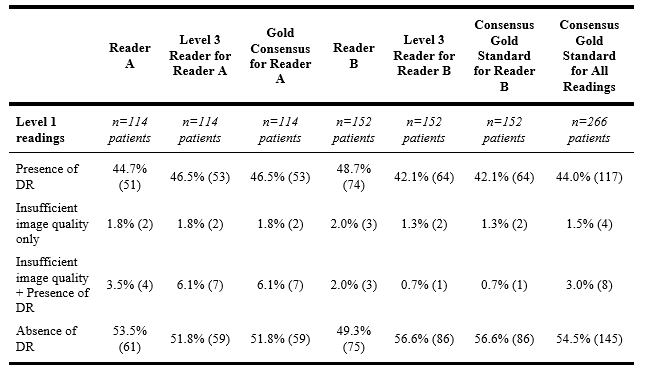

Supplement: Multimedia Appendix 3 [file diabetes_v5i2e17309_app3.PNG]

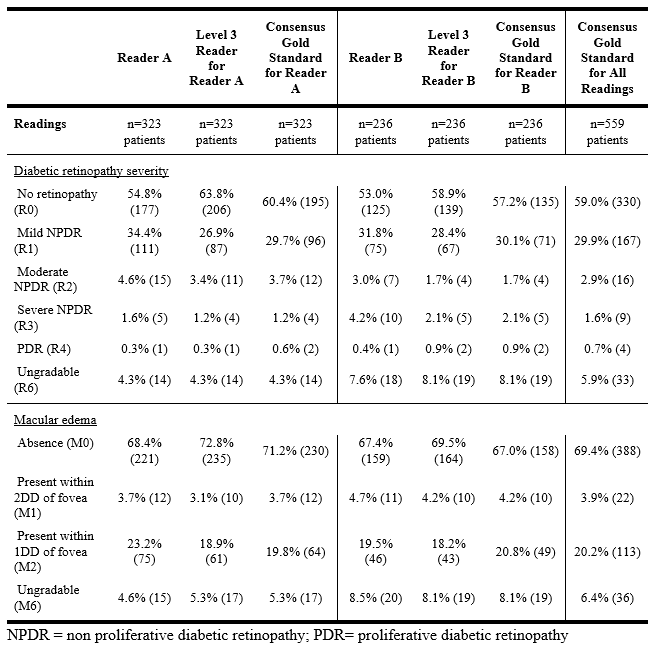

Supplement: Multimedia Appendix 4 [file diabetes_v5i2e17309_app4.PNG]

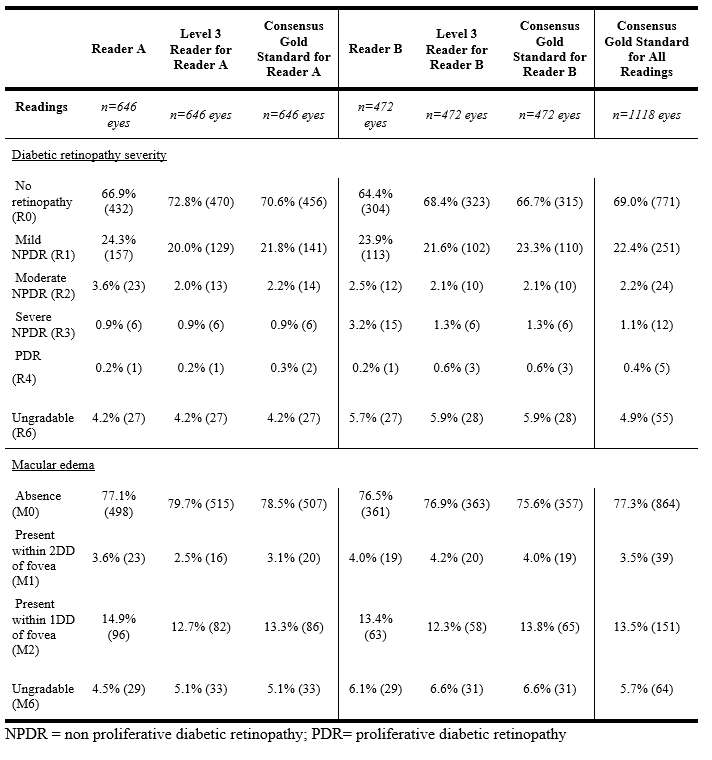

Supplement: Multimedia Appendix 5 [file diabetes_v5i2e17309_app5.PNG]

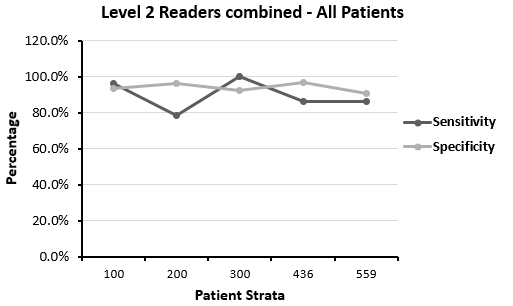

Supplement: Multimedia Appendix 6 [file diabetes_v5i2e17309_app6.PNG]

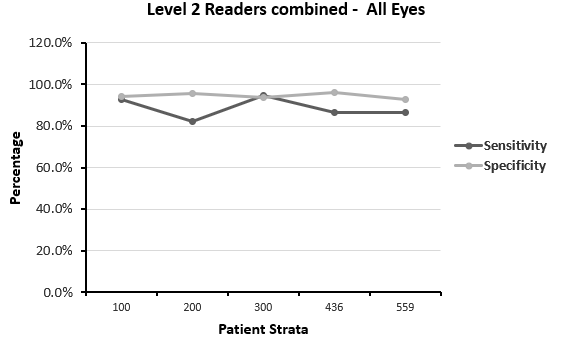

Supplement: Multimedia Appendix 7 [file diabetes_v5i2e17309_app7.PNG]
